# Effect of transcranial direct current stimulation and narrow-band auditory stimulation on the intraoperative electroencephalogram: an exploratoratory feasibility study

**DOI:** 10.3389/fpsyt.2024.1362749

**Published:** 2024-07-16

**Authors:** Oliver G. Isik, Tuan Z. Cassim, Meah T. Ahmed, Matthias Kreuzer, Alice M. Daramola, Paul S. Garcia

**Affiliations:** ^1^ Department of Anesthesiology, Columbia University Vagelos College of Physicians and Surgeons, New York, NY, United States; ^2^ Department of Psychology, School of Social and Behavioral Science, University of Utah, Salt Lake City, UT, United States; ^3^ Sidney Kimmel Medical College, Thomas Jefferson University, Philadelphia, PA, United States; ^4^ Department of Anesthesiology and Intensive Care Medicine, Technical University of Munich School of Medicine and Health, Munich, Germany; ^5^ Department of Anesthesiology, Emory University School of Medicine, Atlanta, GA, United States

**Keywords:** EEG, alpha power, transcranial direct current stimulation, auditory stimulation, neuromodulation

## Abstract

**Introduction:**

During general anesthesia, frontal electroencephalogram (EEG) activity in the alpha frequency band (8–12 Hz) correlates with the adequacy of analgesia. Transcranial direct current stimulation (tDCS) and auditory stimulation, two noninvasive neuromodulation techniques, can entrain alpha activity in awake or sleeping patients. This study evaluates their effects on alpha oscillations in patients under general anesthesia.

**Methods:**

30 patients receiving general anesthesia for surgery were enrolled in this two-by-two randomized clinical trial. Each participant received active or sham tDCS followed by auditory stimulation or silence according to assigned group (TDCS/AUD, TDCS/SIL, SHAM/AUD, SHAM/SIL). Frontal EEG was recorded before and after neuromodulation. Patients with burst suppression, mid-study changes in anesthetic, or incomplete EEG recordings were excluded from analysis. The primary outcome was post-stimulation change in oscillatory alpha power, compared in each intervention group against the change in the control group SHAM/SIL by Wilcoxon Rank Sum testing.

**Results:**

All 30 enrolled participants completed the study. Of the 22 included for analysis, 8 were in TDCS/AUD, 4 were in TDCS/SIL, 5 were in SHAM/AUD, and 5 were in SHAM/SIL. The median change in oscillatory alpha power was +4.7 dB (IQR 4.4, 5.8 dB) in SHAM/SIL, +2.8 dB (IQR 1.5, 8.9 dB) in TDCS/SIL (*p* = 0.730), +5.5 dB in SHAM/AUD (*p* = 0.421), and -6.1 dB (IQR -10.2, -2.2 dB) in TDCS/AUD (*p* = 0.045).

**Conclusion:**

tDCS and auditory stimulation can be administered safely intraoperatively. However, these interventions did not increase alpha power as administered and measured in this pilot study.

## Introduction

Postoperative pain is a nearly universal symptom experienced by patients (1–3), and it has been linked to poor wound healing, lengthier hospital stays, higher healthcare costs, and development of postoperative pain and cognitive disorders (4–7). Providing more adequate analgesia to patients undergoing surgery has potential protective benefits against these complications (8, 9).

Continuous frontal electroencephalography (EEG) monitoring during anesthesia provides metrics that strongly correlate with the adequacy of analgesia, specifically activity in the alpha frequency band (8–12 Hz), thought to reflect thalamocortical oscillations (10–12). Noxious stimulation decreases the strength of alpha oscillations (11, 13), and administration of both opioids and sedatives with analgesic effects increases alpha power (11, 14–16). These observations suggest that alpha power is an objective indicator of the degree of noxious stimulation and the adequacy of analgesia, so techniques of directly boosting this frequency band may have benefits in the intraoperative setting (11, 13).

Recent studies have demonstrated nonpharmacological techniques of neuromodulation, including transcranial direct current stimulation (tDCS) and narrow-band auditory stimulation (17–22). Given the importance of maintaining alpha oscillations in the intraoperative setting to mitigate negative consequences in emergence and recovery post-surgery, it is conceivable that neuromodulation targeting this frequency can be beneficial in multimodal analgesia.

tDCS is an extensively investigated technique of non-invasive brain stimulation that is utilized in a variety of clinical settings including psychiatry, neurology, and pain medicine (23–31). It delivers a battery-powered, low-intensity direct current at 1 to 2 milliamps (mA) via scalp electrodes to the cortical tissue (24). The current flow results in changes to the extracellular *milieu* changing the resting membrane potential of the proximal neurons of the electrode configuration (23–25). Application over the dorsolateral prefrontal cortex is hypothesized to promote thalamocortical oscillations, resulting in the observed increased alpha activity (**Supplementary Figure 1**) (17). Animal models have demonstrated tDCS can quicken emergence and recovery from volatile anesthesia, indicating potential perioperative utility (27, 32). Most recently, a clinical study demonstrated reduced anxiety in patients who received tDCS in the 24 hours prior to surgery (33).

Acoustic stimulation is a form of sensory entrainment capable of modulating EEG patterns: it has been shown to entrain slow EEG waves during sleep (34), and gamma-band synchronization entrained to external 40-Hz sounds has been previously described (35–37). As a modality already studied primarily in the form of music’s effect on perioperative anxiolysis, auditory stimulation can be feasibly administered in an intraoperative setting (38). As auditory stimulation involves thalamocortical communications (**Supplementary Figure 2**), which are thought to be responsible for much of intraoperative frontal alpha power (39), it is possible that auditory stimulation could promote alpha rhythms during general anesthesia.

Despite its potential benefits, neuromodulation has not been explored in the intraoperative setting. This pilot study investigates the feasibility of administering tDCS and narrow-band auditory stimulation, alone and in combination, in the perioperative setting, and their effects on frontal cortical alpha power in patients receiving general anesthesia for surgery. We hypothesized that each intervention would independently and possibly synergistically increase frontal alpha power on EEG after neuromodulation, suggestive of a more adequate state of intraoperative analgesia.

## Methods

Approval for this study was granted by the Institutional Review Board of the Columbia University Irving Medical Center (IRB No. AAAT9632). Written informed consent was obtained from each participant in the study in accordance with the Declaration of Helsinki. This exploratory study was exempt from registration at clinicaltrials.gov as a small feasibility study of a device with prior FDA Investigational Device Exception (IDE). The manuscript was written in accordance with the CONSORT guidelines for the publication of randomized clinical trial data.

### Inclusion & exclusion criteria

Adult patients receiving general anesthesia for surgeries not involving the head, neck, or spine or requiring the use of cardiopulmonary bypass were eligible for participation in this study. Before written consent was obtained, a screening questionnaire was administered to determine the safety of the tDCS intervention. After reaffirming that the patient was not receiving head, neck, or spine surgery, the questionnaire confirmed that the participant had no metal or electronic implants in the brain, skull, or chest; that the participant had no recent history of head trauma with loss of consciousness, that the participant had no severe dermatitis or eczema; that the participant had no history of epilepsy; and that, for female patients, that the participant was not pregnant. Any of the above constituted exclusion criteria for this study. Apart from receiving general anesthesia, no single protocol or technique used to provide analgesia and anesthesia to the patient was specified to the anesthesiology team for study participants. Anesthesiologists chose their anesthetic and analgesic techniques independently of patient involvement in this study.

During surgery, patient participation in the study was terminated if at any point the surgeon, anesthesiologist, or research personnel felt the neuromodulation was unsafe or interfered with the surgery itself or the anesthesiologist’s ability to monitor the patient. In patients who successfully completed the study, those with burst suppression on EEG, change of anesthetic technique between the beginning and end of the study, or incomplete capture of EEG data were excluded from final analysis. Burst suppression and change in anesthetic technique were selected as exclusion criteria to better control the known confounding effect of general anesthesia on potential changes in oscillatory alpha power.

### Procedure

**Figure 1:**
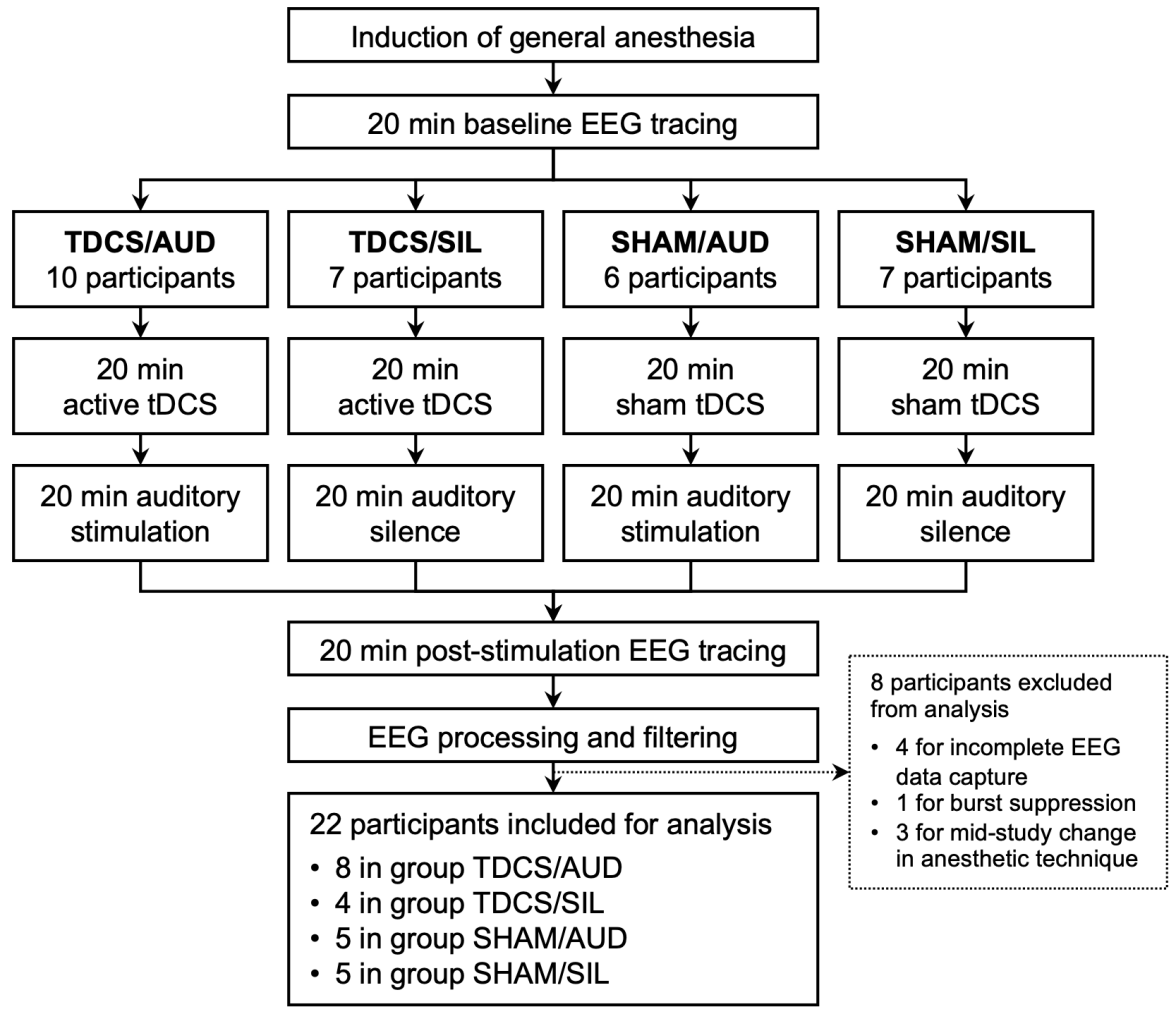
Study flow from induction of general anesthesia to completion of post-neuromodulation EEG tracing by study group, with participant counts during study and after data processing.

30 Patients receiving general anesthesia for surgery at Columbia University Irving Medical Center were recruited for participation in this double-blind, two-by-two randomized clinical trial. After enrollment, participants were randomized to one of four groups. Group TDCS/AUD received active tDCS and auditory stimulation; group TDCS/SIL received active tDCS and auditory silence; group SHAM/AUD received sham tDCS and auditory stimulation; and group SHAM/SIL received sham tDCS and auditory silence (**Figure 1**). These comparisons were preplanned to isolate the individual and combined effects of tDCS and auditory stimulation, based on a hypothesis that their combination would produce a synergistic effect on frontal alpha power.

After induction of general anesthesia, each participant’s baseline frontal EEG was recorded for twenty minutes. After baseline tracings were obtained, twenty minutes of active or sham tDCS were administered followed by twenty minutes of auditory stimulation or silence, according to the participant’s assigned group. Frontal EEG continued to be recorded during stimulation. Following both stimulation techniques, post-stimulation EEG tracings were recorded for an additional twenty minutes. All EEG data were collected before emergence from general anesthesia. After completion of the study, chart review was used to collect important covariates including patient age as well as anesthetic and analgesic medications administered.

### Neuromodulation methods

Standard-definition transcranial direct current stimulation over the dorsolateral prefrontal cortex was delivered at an amplitude of 2 mA using the 1x1 transcranial Electrical Stimulation device, an FDA-approved device by Soterix Medical to employ tDCS in clinical trials. Two 5-cm-by-7-cm foam pads produced by the manufacturer were secured to the patient’s forehead with a strap after lubrication with 8 mL of 0.9% saline solution and served as noninvasive electrodes for administration of tDCS (**Supplementary Figure 3**). For participants receiving sham tDCS, foam pads were still secured, however a pre-designed placebo was administered by the Soterix device. Manufacturer-provided six-digit codes were used to deliver either active or sham tDCS according to the participant’s assigned research group without alerting research personnel to whether the program was administering active or sham stimulation.

Narrow-band auditory stimulation was engineered at 12 Hz and was delivered through external Beats^®^ Bluetooth headphones placed over the patient’s ears (**Supplementary Figure 3**). Audio was administered from the research personnel’s phone, with tracks de-identified and either set to play 20 minutes of either the recorded track or silence. Volume was preset for a peak stimulation intensity of 80 dB in active auditory stimulation to ensure consistency and safety.

Patient baseline and post-stimulation EEG was captured using the Masimo Sedline™ Sedation Monitor’s frontal EEG sensor. This four-channel frontal EEG montage is used to guide patient sedation under general anesthesia. The monitor provides real-time interpretation of the patient’s EEG to clinicians, including raw EEG, spectrograms, and commonly analyzed parameters like the spectral edge frequency and Patient State Index™. This information was viewable to the anesthesiologist and research team during the study, and the anesthesiologist was warned that during tDCS or auditory stimulation, the neuromodulation may alter the EEG waveform artificially. The Sedline™ monitor records EEG tracings at a capture rate of 178 Hz and saves them for data extraction. These EEG tracings were collected for each study participant on a secure USB drive for analysis.

### Data and statistical analysis

After excluding data from participants with burst suppression, changes in anesthetic technique, and incomplete data capture, EEG tracings from the more central L1 and R1 electrodes (**Supplementary Figure 3**) were processed using a fifth-order bandpass filter between 0.5 and 30 Hz and removed of impulse artifacts to generate five minutes of artifact-free EEG during each phase of the study for each participant. These electrodes were chosen as the lateral L2 and R2 had greater artifact burden. Density spectral array (DSA) spectrograms were created for each participant during each study phase. Power spectral densities (PSDs) were generated with 95 percent confidence intervals for the power density at each frequency for each study participant before and after stimulation, then averaged among participants within each study group.

The outcome analyzed in the study was the change in oscillatory alpha power after neuromodulation for each participant. A commonly employed metric to measure alpha activity, oscillatory alpha power is calculated by measuring the increase in EEG power in the alpha band relative to the adjacent theta (3.5–7.5 Hz) and beta (20–30 Hz) bands (40). Median changes and interquartile ranges for each study group were calculated. The effects of tDCS and auditory stimulation, alone and in combination, were evaluated by comparing changes in oscillatory alpha power in groups TDCS/AUD, TDCS/SIL, and SHAM/AUD to group SHAM/SIL using Wilcoxon Rank Sum tests.

Due to the small sample size of this pilot study, statistical tests did not adjust for potential confounding effects, however several demographic covariates, medical conditions, and variables known to influence the presence alpha oscillations were compared qualitatively across study groups: age; sex; American Society of Anesthesiologists (ASA) Physical Status; presence of comorbidities; and technique of general anesthesia (volatile, total intravenous anesthesia, or mixed), and whether the patient received additional opioid or non-opioid analgesia boluses during their participation in the study. No hypothesis testing was performed on these covariates. Age and sex were obtained from the patient’s chart, ASA Physical Status and comorbidities were obtained from the anesthesia preoperative evaluation, and both anesthetic technique and analgesic medication administration were obtained from the intraoperative anesthesia record.

## Results

Of the 30 participants enrolled in this clinical trial, 10 were in group TDCS/AUD, 7 were in TDCS/SIL, 6 were in SHAM/AUD, and 7 were in SHAM/SIL. Of these, 4 participants were excluded from analysis for incomplete EEG data capture, 1 was excluded for burst suppression during the study, and 3 were excluded due to a change in anesthetic strategy during the study period. No participants were excluded due to unsuccessful administration of neuromodulation, a need to terminate study participation from intraoperative safety concerns, or interference with the procedure or anesthetic monitoring. Of the 22 participants included for final analysis, 8 were in group TDCS/AUD, 4 were in TDCS/SIL, 5 were in SHAM/AUD, and 5 were in SHAM/SIL.

### Characteristics of participants by study group

Demographic and anesthetic comparisons among participants in different study groups are provided in **Table 1**. Participants were slightly younger in group SHAM/AUD, however interquartile ranges of age among the four groups were comparable. Participants in group TDCS/AUD were more likely to have a higher ASA Physical Status, though the prevalence of neuropsychiatric comorbidities was comparable across all groups. In terms of anesthetic technique, more participants in group SHAM/SIL received purely inhalational anesthesia during maintenance, and more participants in groups SHAM/AUD and SHAM/SIL received additional boluses or infusions of analgesic medication during the study.

**Table 1:** Characteristics of participants in each study group.

	TDCS/AUD *n* = 8	TDCS/SIL *n* = 4	SHAM/AUD *n* = 5	SHAM/SIL *n* = 5
Median Age (IQR), years	58 (53, 61)	56 (45, 67)	40 (40, 63)	64 (40, 65)
Sex Male	5 (63%)	2 (50%)	3 (60%)	2 (40%)
ASA Physical Status				
1	0 (0%)	1 (25%)	1 (20%)	0 (0%)
2	3 (38%)	2 (50%)	3 (60%)	3 (60%)
3	5 (63%)	1 (25%)	1 (20%)	2 (40%)
Comorbid Conditions				
Pulmonary	7 (88%)	3 (75%)	3 (60%)	2 (40%)
Cardiovascular	6 (75%)	2 (50%)	2 (40%)	3 (60%)
Neurologic	1 (13%)	0 (0%)	1 (20%)	1 (20%)
Psychiatric	2 (25%)	2 (50%)	0 (0%)	2 (40%)
Renal	2 (25%)	1 (25%)	2 (40%)	0 (0%)
Gastrointestinal/Hepatic	5 (63%)	1 (25%)	2 (40%)	3 (60%)
Endocrine	5 (63%)	2 (50%)	2 (40%)	2 (40%)
Hematologic	4 (50%)	1 (25%)	1 (20%)	2 (40%)
Oncologic	2 (25%)	2 (50%)	0 (0%)	0 (0%)
Anesthetic Technique				
Inhalational	5 (63%)	2 (50%)	3 (60%)	5 (100%)
TIVA	1 (13%)	0 (0%)	0 (0%)	0 (0%)
Mixed	2 (25%)	2 (50%)	2 (40%)	0 (0%)
Analgesic Redosed	3 (38%)	1 (25%)	4 (80%)	3 (60%)

TIVA, total intravenous anesthesia.

### EEG spectra and alpha power after neuromodulation

**Figure 2:**
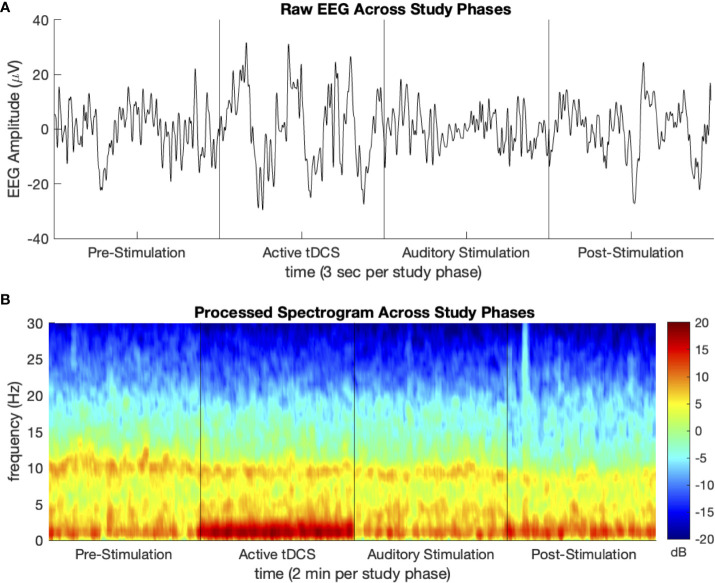
Sample **(A)** processed EEG tracing and **(B)** density spectral array (spectrogram) for one study participant at each phase of the study. Participant group TDCS/AUD.

**Figure 3:**
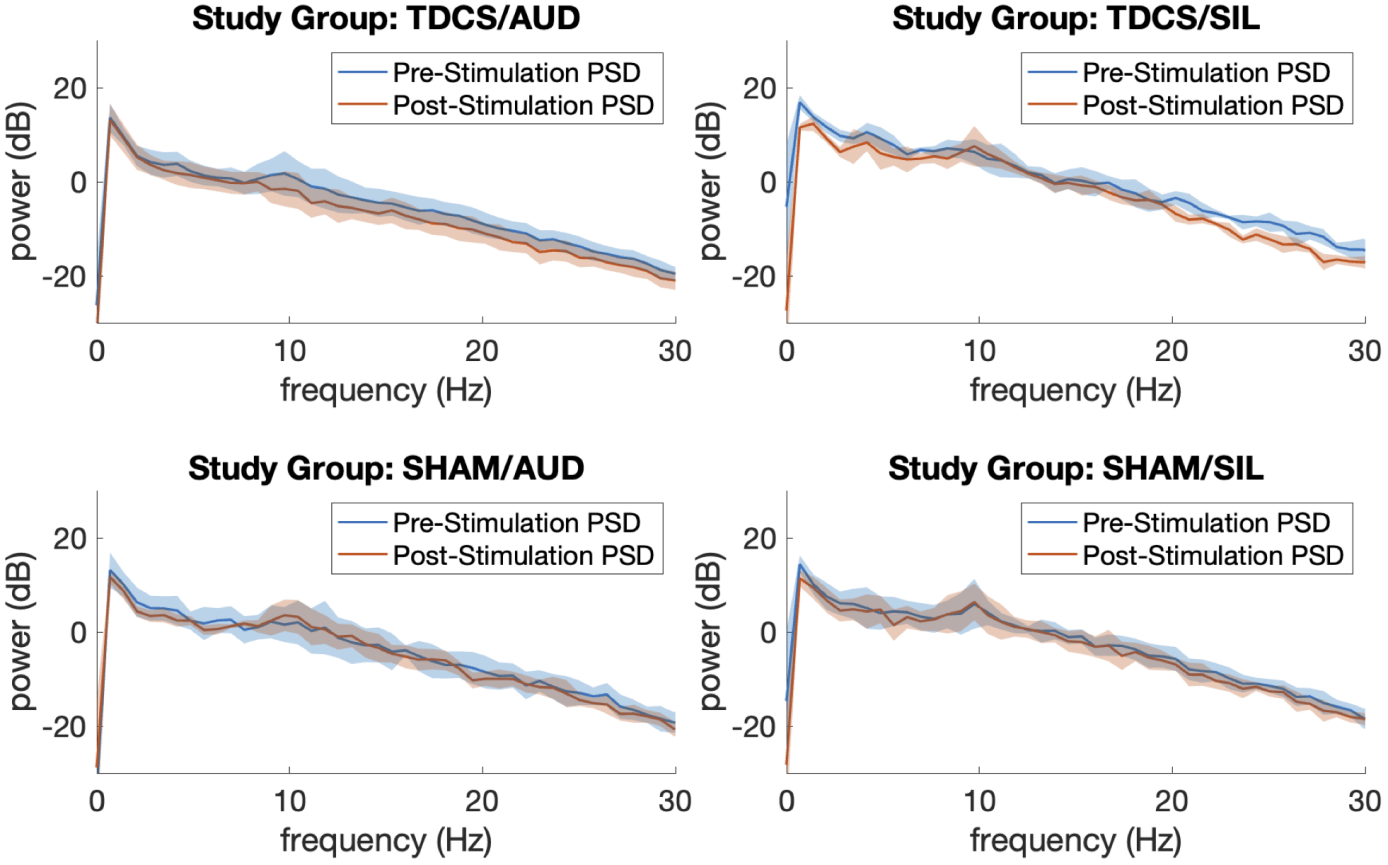
Average power spectral densities by study group before and after neuromodulation with 95% confidence intervals.

Segments of artifact-free EEG tracings and processed spectrograms of one study participant at each phase of the study are shown in **Figure 2**. This participant was assigned to Group TDCS/AUD. Noise from active tDCS results in the increased activity in the delta (1–4 Hz) frequency band during that phase of the study. The PSDs with 95 percent confidence intervals before and after stimulation, averaged over participants in each group of the study are depicted in **Figure 3**. Across all four study groups, minimal changes were seen in the average PSDs before and after stimulation.

**Figure 4:**
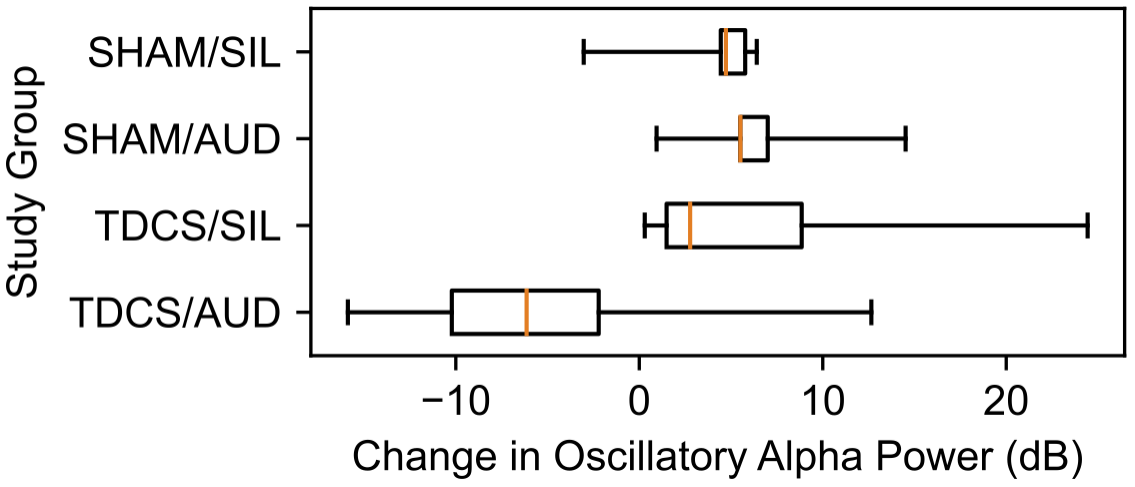
Change in oscillatory alpha power, in decibels, after neuromodulation by study group.

A boxplot of changes in oscillatory alpha power by study group is provided in **Figure 4**. In the control group SHAM/SIL, the median change in oscillatory alpha power was +4.7 dB (IQR 4.4, 5.8 dB). In the group receiving only auditory stimulation, SHAM/AUD, the median change was +5.5 dB (IQR 5.5, 7.0 dB; *p* = 0.421). In the group receiving only tDCS, TDCS/SIL, the median change was +2.5 dB (IQR 1.5, 8.9 dB; *p* = 0.730). In the group receiving tDCS and auditory stimulation in combination, TDCS/AUD, the median change was -6.1 dB (IQR -10.2, -2.2 dB; *p* = 0.045).

## Discussion

Given that every enrolled participant was able to successfully complete the protocol without safety concerns from the surgical team, anesthesiology team, or research team, this study demonstrates that tDCS and narrow-band auditory stimulation can be administered safely to patients receiving general anesthesia during surgery. That said, as patients were not followed postoperatively, no guarantee against complications can be given based on these data. That complications are known and minimal after administration of tDCS and auditory stimulation in awake patients suggests similar rates after receiving these interventions intraoperatively, however this study cannot address that question.

Analysis of EEG spectra showed that active tDCS and auditory stimulation, alone and in combination, did not visibly increase oscillatory alpha power in this study. Although the combination of tDCS and auditory stimulation produced a lower change in oscillatory alpha power than no neuromodulation at all, the fact that this result was seen without any observable decrease in alpha power after either form of neuromodulation alone suggests that the observed decrease may be due to unmeasured confounding.

Given that each of these techniques achieves neuromodulation in awake and sleeping patients (17–22, 34–37), it is reasonable to conclude that their effects in patients under general anesthesia for surgery may be modest when compared to the influence of age, comorbidities, noxious stimulation during surgery, and pharmacologic strategy. While alpha power is a putative mechanism of action which is very much linked to pain, other factors might be at play, such as changes in excitability, connectivity, or blood flow. The stimulation threshold may be higher in patients under general anesthesia, as the pharmacologic agents themselves are profound neuromodulators. In this study, auditory stimulation was administered at close to the upper threshold of what is accepted as a safe decibel level, but tDCS intensities can vary greatly. While this study employed tDCS at 2 mA, a common selection, higher intensities may be necessary to induce the desired neuromodulation in patients under anesthesia.

The small sample size, broad inclusion criteria, and inherent design in this pilot study limit the ability to control for potential confounding effects. Furthermore, the randomization protocol in this study did not result in equal group sizes. While age ranges were similar among study groups (**Table 1**), median age was lower in the SHAM/AUD group, and the TDCS/AUD group had a greater prevalence of comorbid disease, as evidenced by the substantially higher rates of ASA 3 Physical Status (**Table 1**). As age has a known correlation with alpha power (41), and comorbidities can influence the physiologic response to any intervention, future larger and more balanced studies investigating benefits of intraoperative neuromodulation will need to control for these potential confounding effects.

Additionally, noxious stimulation is known to have a potent influence on alpha power (11, 13). Without controlling for the precise type of surgery, it is difficult to account for this effect. Moreover, pre-stimulation baseline tracings were often recorded between induction of general anesthesia and first surgical incision, whereas post-stimulation tracings were recorded mid-procedure. This profound difference in degree of stimulation, even though it was present across all four study groups, may have masked any observable effect of tDCS and auditory stimulation on alpha activity, decreasing study power.

Along with noxious stimulation, administration of analgesic medication improves alpha power (11). The protocol for how to dose analgesic medication is not standardized in anesthesia (42), nor was it specified to anesthesiologists during this study. As is seen in **Table 1**, participants who received sham tDCS were more likely to have received a bolus of analgesia than participants who received active tDCS. Although the small sample size in this study precluded the investigation of any potential confounder via a statistical model, it can be noted that such a difference may specifically mask the beneficial effect of tDCS.

The effects of intraoperative pharmacologic decision-making must also be considered. Different anesthetic strategies, as well as different doses of each individual hypnotic or analgesic drug, can influence alpha power (14, 16). Such variability can significantly influence this study’s observed result. In several cases, the anesthesia team prepared for emergence by switching from maintenance with a volatile anesthetic to a propofol infusion before this study’s EEG recording was completed. Additionally, one patient received an excessive dose of sedative-hypnotic agent, and burst suppression was witnessed. Apart from excluding these specific cases from analysis, this study had limited control for differing anesthetic techniques. Future research into the effects of nonpharmacologic neuromodulation will have to standardize anesthetic technique, both in managing level of sedation and analgesia. Indeed, given that different anesthetic and analgesic agents produce different neuromodulation according to their pharmacologic mechanisms of action—opioid receptor agonism by opioids; GABA agonism by propofol, etomidate, volatiles, or benzodiazepines; NMDA antagonism by ketamine; alpha_2_ agonism by dexmedetomidine—future research may well find that these nonpharmacologic neuromodulation techniques are more effective when combined with a particular anesthetic, producing more synergistic neuromodulation.

Perhaps a final limiting feature of the study’s ability to detect the benefit of tDCS was the nature in which the neuromodulation was administered. This study employed standard-definition tDCS, which utilizes large 5-cm-by-7-cm moistened foam electrodes on either side of the forehead (**Supplementary Figure 3**). High-definition tDCS (HD-tDCS) is a technique using much smaller gel-based electrodes at precisely chosen sites, which if applied correctly, have the potential to target brain structures of interest much more specifically (43). Similarly, transcranial alternating current stimulation (tACS), which delivers alternating current at a specified frequency, may also offer a route to entrain specific EEG frequencies. Intraoperatively, alpha frequencies may be entrained, while postoperatively, higher frequency beta and gamma (30- to 40-Hz) waves may be augmented to potentially enhance recovery. These two variants of the tDCS technique explored in this study may also prove to be valuable techniques of nonpharmacologic neuromodulation in the intraoperative setting.

## Conclusions

In this pilot study, transcranial direct current stimulation and narrow-band auditory stimulation were safe and feasible to administer in the intraoperative setting. Their benefits on frontal alpha power are more difficult to elicit under a state of general anesthesia than in an awake state. Further research investigating the potential utility of these interventions in patients receiving general anesthesia will need larger sample sizes, better control for pharmacologic technique and noxious stimulation, and an investigation of different intensities of neuromodulation.

## Data availability statement

The raw data supporting the conclusions of this article will be made available by the authors, without undue reservation.

## Ethics statement

The studies involving humans were approved by Columbia University Irving Medical Center. The studies were conducted in accordance with the local legislation and institutional requirements. The participants provided their written informed consent to participate in this study.

## Author contributions

OI: Conceptualization, Data curation, Formal analysis, Funding acquisition, Investigation, Methodology, Project administration, Resources, Software, Supervision, Validation, Visualization, Writing – original draft, Writing – review & editing. TC: Conceptualization, Data curation, Formal analysis, Investigation, Methodology, Project administration, Resources, Software, Supervision, Validation, Visualization, Writing – original draft, Writing – review & editing. MA: Conceptualization, Data curation, Formal analysis, Investigation, Methodology, Project administration, Resources, Software, Supervision, Validation, Visualization, Writing – original draft, Writing – review & editing. MK: Formal analysis, Methodology, Writing – original draft, Writing – review & editing, Project administration, Resources, Software, Supervision, Validation. AD: Conceptualization, Investigation, Methodology, Resources, Writing – original draft, Writing – review & editing. PG: Conceptualization, Data curation, Formal analysis, Funding acquisition, Investigation, Methodology, Project administration, Resources, Software, Supervision, Validation, Visualization, Writing – original draft, Writing – review & editing.
