# Supplementary material for: Effect of transcranial direct current stimulation and narrow-band auditory stimulation on the intraoperative electroencephalogram: an exploratoratory feasibility study
Source: Front Psychiatry. 2024 Jul 16;15:1362749. doi: 10.3389/fpsyt.2024.1362749 (PMC11286499; doi:10.3389/fpsyt.2024.1362749)
Supplement: Supplementary file 1 [file DataSheet_1.docx]

**Effect of transcranial direct current stimulation and narrow-band auditory stimulation on the intraoperative electroencephalogram: an exploratory feasibility study**

**Supplementary Material**

**eFigure 1**. Heat map of induced electric fields in brain tissue by standard-definition tDCS applied over the dorsolateral prefrontal cortex. White circles indicate the location of the axial, coronal, and sagittal slices in the brain.

**eFigure 2**. Neural pathway of auditory stimulation from cochlea through thalamic nuclei and finally to primary auditory cortex.

**eFigure 3.** Placement of transcranial direct current stimulation (tDCS) foam electrodes (red, blue) with strap (gray) to secure them to the patient’s forehead, headphones (black) for delivery of narrow-band auditory stimulation, and Sedline™ sensor (white) for continuous frontal EEG monitoring.
